# Isoflurane anesthesia disrupts the cortical metabolome

**DOI:** 10.1152/jn.00375.2020

**Published:** 2020-10-28

**Authors:** Aaron G. Baer, Allen K. Bourdon, Joshua M. Price, Shawn R. Campagna, Daniel A. Jacobson, Helen A. Baghdoyan, Ralph Lydic

**Affiliations:** ^1^Department of Anesthesiology, University of Tennessee Medical Center, Knoxville, Tennessee; ^2^Department of Chemistry, University of Tennessee, Knoxville, Tennessee; ^3^Office of Information Technology, University of Tennessee, Knoxville, Tennessee; ^4^Biological and Small Molecule Mass Spectrometry Core, University of Tennessee, Knoxville, Tennessee; ^5^Department of Psychology, University of Tennessee, Knoxville, Tennessee; ^6^Oak Ridge National Laboratory, Oak Ridge, Tennessee

**Keywords:** in vivo microdialysis, liquid chromatography-dual mass spectrometry, prefrontal cortex, states of consciousness, untargeted metabolomics

## Abstract

Identifying similarities and differences in the brain metabolome during different states of consciousness has broad relevance for neuroscience and state-dependent autonomic function. This study focused on the prefrontal cortex (PFC) as a brain region known to modulate states of consciousness. Anesthesia was used as a tool to eliminate wakefulness. Untargeted metabolomic analyses were performed on microdialysis samples obtained from mouse PFC during wakefulness and during isoflurane anesthesia. Analyses detected 2,153 molecules, 91 of which could be identified. Analytes were grouped as detected during both wakefulness and anesthesia (*n* = 61) and as unique to wakefulness (*n* = 23) or anesthesia (*n* = 7). Data were analyzed using univariate and multivariate approaches. Relative to wakefulness, during anesthesia there was a significant (*q* < 0.0001) fourfold change in 21 metabolites. During anesthesia 11 of these 21 molecules decreased and 10 increased. The Kyoto Encyclopedia of Genes and Genomes database was used to relate behavioral state-specific changes in the metabolome to metabolic pathways. Relative to wakefulness, most of the amino acids and analogs measured were significantly decreased during isoflurane anesthesia. Nucleosides and analogs were significantly increased during anesthesia. Molecules associated with carbohydrate metabolism, maintenance of lipid membranes, and normal cell functions were significantly decreased during anesthesia. Significant state-specific changes were also discovered among molecules comprising lipids and fatty acids, monosaccharides, and organic acids. Considered together, these molecules regulate point-to-point transmission, volume conduction, and cellular metabolism. The results identify a novel ensemble of candidate molecules in PFC as putative modulators of wakefulness and the loss of wakefulness.

**NEW & NOTEWORTHY** The loss of wakefulness caused by a single concentration of isoflurane significantly altered levels of interrelated metabolites in the prefrontal cortex. The results support the interpretation that states of consciousness reflect dynamic interactions among cortical neuronal networks involving a humbling number of molecules that comprise the brain metabolome.

## INTRODUCTION

Among the most daunting unanswered questions in neuroscience are those that concern the mechanisms by which states of consciousness are generated. The role of the prefrontal cortex (PFC) as a modulator of states of consciousness is undergoing active investigation from an electrophysiology and connectivity perspective (Carlén 2017; Cowan et al. 2020; Joglekar et al. 2018; Krone et al. 2020; Pal et al. 2018; van Vugt et al. 2018). Although the PFC has long been known to modulate states of consciousness (Muzur et al. 2002), we are aware of only one study in mice that compared the PFC metabolome during wakefulness and sleep (Bourdon et al. 2018).

The brain metabolome comprises thousands of low-molecular-weight molecules that underlie fundamental cell biology (Alonso et al. 2015) and have wide-ranging relevance for basic and clinical neurophysiology (Patti et al. 2012). Brain site-specific features of the metabolome (Gonzalez-Riano et al. 2016) can be revealed by analyzing microdialysis samples of extracellular fluid (Bongaerts et al. 2018) using ultrahigh-performance liquid chromatography-high-resolution mass spectrometry (UHPLC-HRMS).

Sleep and anesthesia are different states of consciousness that exhibit similar traits such as altered autonomic regulation, motor hypotonia, and state-specific changes in the electroencephalogram (Akeju and Brown 2017; Baghdoyan and Lydic 2012; Brown et al. 2010; Garrity et al. 2015; Horner et al. 2014; Lydic and Baghdoyan 2005, Lydic et al. 2018; Vanini et al. 2020). The brain mechanisms generating states of wakefulness, sleep, and anesthesia are not fully understood. The similarities between sleep and anesthesia support the view that a mechanistic understanding of sleep or anesthesia will be reciprocally informative. Evidence is available regarding the PFC metabolome during sleep (Bourdon et al. 2018), but we are aware of no data quantifying the effect of isoflurane anesthesia on the PFC metabolome of mice. Therefore, the goal of the present study was to use untargeted, discovery-based metabolomics to compare the PFC metabolome of C57BL/6J mice during wakefulness and during the isoflurane-induced loss of wakefulness.

The present study collected microdialysis samples from mouse PFC during wakefulness and during isoflurane anesthesia and analyzed these samples using UHPLC-HRMS. By holding species, sex, and brain region constant relative to previous studies (Bourdon et al. 2018), it was possible to directly compare the PFC metabolome during anesthesia and sleep. The results reveal more differences than similarities between the PFC metabolome during anesthesia and sleep (Bourdon et al. 2018). During isoflurane anesthesia, the PFC metabolome differed significantly from the waking metabolome for neurotransmitter precursors that regulate point-to-point chemical transmission (Zhang et al. 2020) and for molecules that modulate volume transmission (Marcoli et al. 2015). The results provide novel comparisons of metabolites and their biofunction during the loss of wakefulness caused by sleep (Bourdon et al. 2018) and by isoflurane anesthesia.

## MATERIALS AND METHODS

### 

#### Animals and animal care.

Adult male C57BL/6J mice (B6, stock no. 000664; *n* = 24) were purchased from the Jackson Laboratory (Bar Harbor, ME). These mice comprised a different group from those used for previous studies of the PFC metabolome during sleep and wakefulness (Bourdon et al. 2018) and from those used for quantification of PFC neurotransmitters during isoflurane anesthesia (Zhang et al. 2020). Throughout the study, mice had ad libitum access to food (Teklad 8640) and water. Features of mouse housing included a 12-h:12 h light-dark cycle (lights on from 7:00 AM to 7:00 PM) with room temperature (average 23°C) and humidity (average 45%) controlled. Environmental conditions were monitored 24 h/day via WiFi connection between mouse room sensors and cell phones of the investigators. Mice were maintained in good health throughout the study and were inspected daily by laboratory staff and weekly by the University’s Office of Laboratory Animal Care. All studies were reviewed and approved by the University of Tennessee Institutional Animal Care and Use Committee and adhered to the National Institutes of Health’s *Guide for the Care and Use of Laboratory Animals* (National Academies Press, 8th ed., Washington, DC, 2011).

#### In vivo microdialysis and experimental design.

**Fig. 1.**
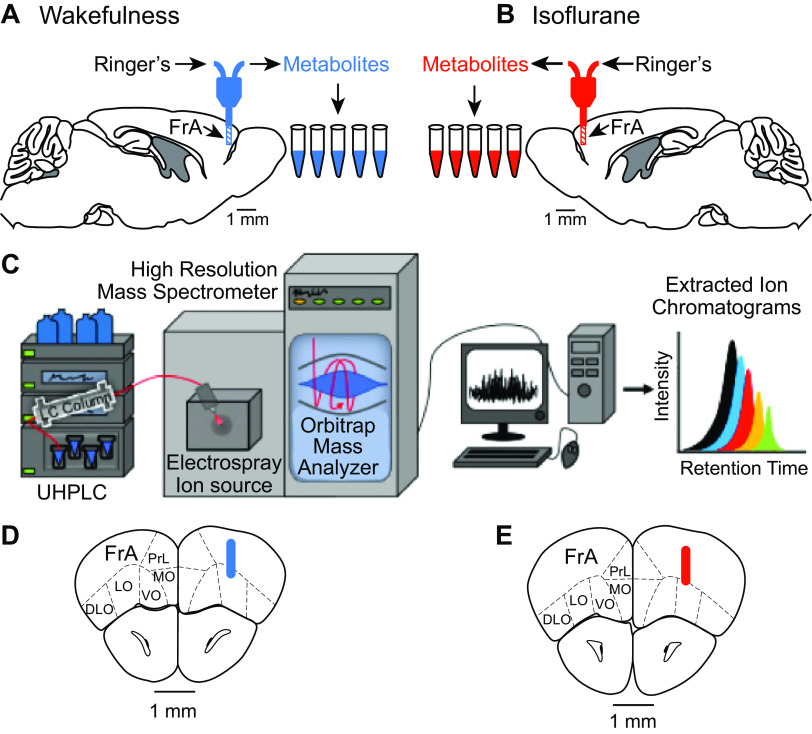
Schematic of experimental design and histological analyses. The design made it possible to obtain microdialysis samples from prefrontal cortex of 12 intact, behaving mice during wakefulness (*A*) and 12 intact mice anesthetized with isoflurane (*B*). Sagittal sections of mouse brain were modified from (Franklin and Paxinos 2008). Five microdialysis samples were obtain from each of the 24 mice. *C*: samples were frozen at −80°C and subsequently analyzed using ultrahigh-performance liquid chromatography-high resolution mass spectrometry (UHPLC-HRMS; modified from Lydic et al. 2018). Seven to ten days after each experiment, mice were deeply anesthetized, and brains were processed for histology to localize dialysis sites. Colored cylinders represent the average location of the dialysis membranes during wakefulness (blue; *D*) and isoflurane anesthesia (red; *E*) and are drawn to scale on coronal sections from (Franklin and Paxinos 2008). FrA, frontal association cortex; DLO, dorsolateral orbital cortex; LO, lateral orbital cortex; MO, medial orbital cortex; PrL, prelimbic cortex; VO, ventral orbital cortex.

This study used a completely randomized design. Microdialysis samples were collected from the PFC during wakefulness (Fig. 1*A*; *n* = 12 mice) or during isoflurane anesthesia (Fig. 1*B*; *n* = 12 mice). The stereotaxic aim point was 2.6 mm anterior, 1 mm lateral to the midline, and 1 mm ventral, relative to bregma (Franklin and Paxinos 2008). As described previously (Zhang et al. 2020), mice were anesthetized with isoflurane, and an implanted guide tube was secured to the skull with dental acrylic. Mice recovered from the surgery for 1 wk and then were acclimated to being handled and to being placed in an open glass recording chamber. On the day of the experiment, a CMA model 7 microdialysis probe (cuprophane membrane:1 mm long, 0.24 mm diameter, 6 kDa cutoff; CMA, Holliston, MA) was inserted into the guide tube and was perfused continuously with Ringer’s solution (NaCl 147 mM, CaCl_2_ 1.2 mM, KCl 2.7 mM, MgCl_2_ 0.85 mM; CMA) at a flow rate of 1 μL/min using a CMA 4004 microdialysis pump. Beginning 30–40 min after probe insertion into the brain, five microdialysis samples (each 25 μL) were collected sequentially on ice from each mouse. For the waking condition, the pump and dialysis probe were connected via a liquid swivel (model 375/D/22QM, Instech, Plymouth Meeting, PA), making it possible for the mouse to move freely during sample collection. The behavior of each mouse was monitored to ensure that samples were collected during wakefulness.

For the isoflurane experiments, after a mouse had been anesthetized, the dialysis probe was lowered into the PFC, and delivered isoflurane concentration was maintained at 1.3% for the duration of the 125-min microdialysis sample collection period. A concentration of 1.3% isoflurane corresponds to the minimum alveolar concentration, or EC_50_, for the B6 mouse (Sonner et al. 2000). Delivered isoflurane concentration was measured continuously using spectrophotometry (Cardiocap/5, Datex-Ohmeda). Core body temperature was maintained at 36–37°C. Delivered isoflurane concentration, core body temperature, and respiratory rate were recorded every 10 min. These measurements were stable throughout the experiments. Microdialysis samples were collected into 0.6-mL centrifuge tubes on ice held in a Styrofoam cooler. All microdialysis samples were stored at −80°C before metabolite quantification.

**Fig. 2.**
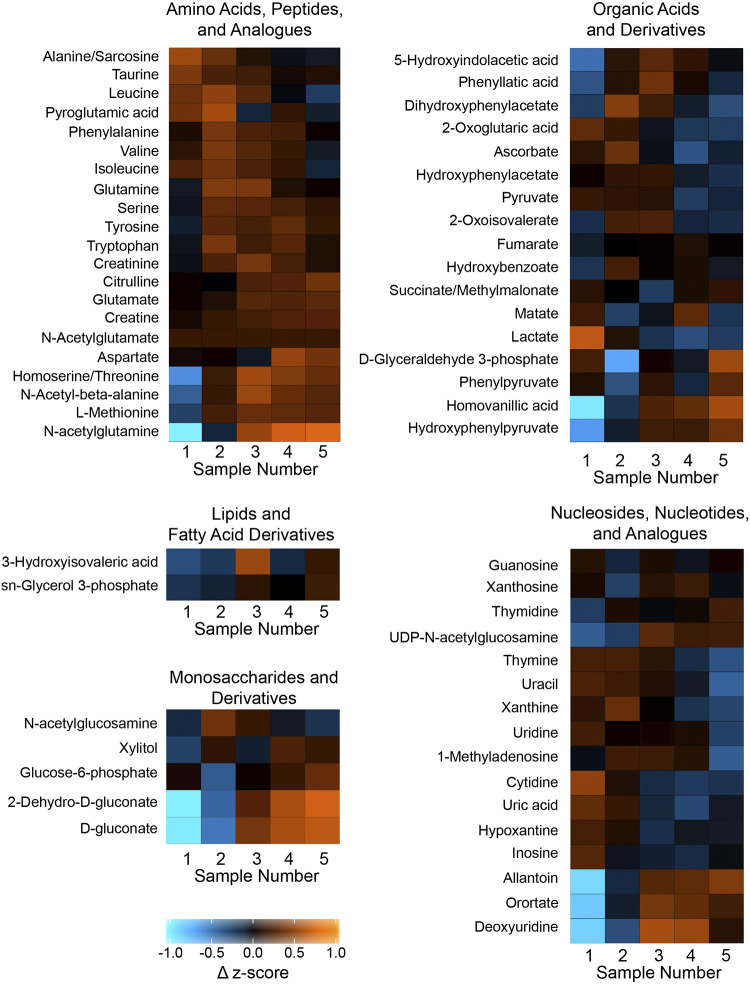
Heat maps illustrating changes in prefrontal cortex metabolites during isoflurane anesthesia compared with wakefulness, with colors representing changes in *z*-score of metabolite ion intensities. Metabolites listed to the *left* of each heat map are organized into 5 chemical groups. Sample number below the heat map indicates each 25-min microdialysis interval. The scale at *bottom left* ranges from −1.0 to +1.0, where color represents changes in *z*-score ranging from negative to positive 1. Quantitative data for each of the 61 molecules represented in this figure are presented in Supplemental Table S1 (https://figshare.com/s/42669ca81d5079678004). Heat maps were generated in R using ggplot2.

Microdialysis sample collection typically reveals time-dependent changes in levels of analytes that we have shown may vary by molecule (Zhang et al. 2020) and brain region (Van Dort et al. 2009). For both the wakefulness and anesthesia conditions, all microdialysis samples were collected between 10:00 AM and 2:00 PM. Onset time and duration of microdialysis sample collection were standardized across the wakefulness and anesthesia experiments. The absence of time-dependent confounds is illustrated by the heatmaps of Fig. 2.

#### Metabolite quantification.

Dialysis samples were analyzed using a metabolomics method described previously (Bourdon et al. 2018; Stough et al. 2016). Instrumentation (Fig. 1*C*) for UHPLC-HRMS included an Ultimate 3000 LC pump in tandem with an Exactive Plus benchtop Orbitrap mass spectrometer from Thermo Fisher Scientific. For UHPLC, the mobile phase comprised 10 mM tributylamine in 97:3 HPLC-grade water-methanol at pH 3.0. Injection volume was 5 µL, and there was no sample pretreatment before injection. Flow rate was 200 µL/min, and the gradient comprised *solvent A*, consisting of 97:3 HPLC-grade water-methanol, 10 mM tributylamine, and 15 mM acetic acid. *Solvent B* was HPLC-grade methanol. The mobile phase gradient from 0 to 5 min was 0% *B*, from 5 to 13 min was 20% *B*, from 13 to 15.5 min was 55% *B*, from 15.5 to 19 min was 95% *B*, and from 19 to 25 min was 0% *B*. With the exception of injection volume, the forgoing procedures were identical to those described previously (Stough et al. 2016). Online chromatography proceeded with a Synergi Hydro-RP column (100 × 2.1 mm, 2.5 µm, 100 Å) using a previously reported gradient (Lu et al. 2010). For HRMS, full scan analysis in negative mode from 80 to 1,000 *m/*z was accomplished during a 25-min run time. Area under the curve data were integrated using Metabolomic Analysis and Visualization Engine (MAVEN) software. Exact mass and retention times of identified metabolites were confirmed using previously analyzed standards purchased from Fisher Scientific, Inc. Orbitrap mass analyzer technology confirmed reliable metabolite identification via measurements of exact mass and retention time (Lu et al. 2010; Sporns et al. 2005). Quality control samples comprised of amino acids and other small molecules were analyzed weekly to ensure conservation of analyte relative abundances and retention times.

#### Histological analysis of dialysis sites.

Seven days after microdialysis sample collection, the mice were deeply anesthetized and decapitated. Brains were removed and soak-fixed in 10% formalin. Serial coronal sections were cut at 35 μm thickness, stained with Perl’s DAB for probe tract detection, and counterstained with thionine to reveal cell bodies. The stereotaxic position of a microdialysis probe within the PFC (Fig. 1, *D* and *E*) was confirmed by comparing the stained tissue sections with the coordinates of a mouse brain atlas (Franklin and Paxinos 2008).

#### Statistical analyses.

This study adhered to the criterion that, to be included in the data set, metabolites must be detected in 80% of the 60 dialysis samples collected during wakefulness and in 80% of the 60 samples collected during isoflurane anesthesia. This criterion required that neither the samples collected during wakefulness nor the samples collected during anesthesia could be missing more than 20% of a metabolite. Sixty-one metabolites were collected during both wakefulness and anesthesia and are referred to as shared metabolites. The product of 61 metabolites in 120 microdialysis samples created a data matrix of 7,320 points. Within that matrix, there were 24 missing values, which were replaced with the nearest-neighbor average for a specific metabolite and mouse. Multivariate and univariate analyses were performed on a data set consisting of 7,320 metabolite measures. As described below in results, these analyses confirmed the detection of 2,153 molecules. The Shapiro–Wilk test for normality and Levene’s test of equality of variances were used to evaluate the mixed-model ANOVA modeling assumptions (Littell et al. 2006). Box plots and studentized residuals were used to check for outliers. Multiple dependent measures resulted in unequal variances across treatment groups, lack of normally distributed residuals, and outliers. Therefore, a mixed-model ANOVA on ranks was used to analyze for treatment differences among the 61 shared metabolites (Conover 1999; Littell et al. 2006). Benjamini Hochberg *P*-value adjustment was applied across the 61 shared metabolites to adjust for the number of statistical tests performed. Mixed-model ANOVA was performed using SAS software and the GLIMMIX procedure (SAS Institute 2019).

In addition to mixed-model ANOVA, multivariate techniques were performed to determine how similar or different the relative concentrations of the 61 shared metabolites were between states of wakefulness and isoflurane anesthesia. A log transform was performed on the data before a partial least squares discriminate analysis (PLS-DA) was conducted to stabilize heteroscedasticity (van den Berg et al. 2006). The explained variance in the *Y* matrix (*R*^2^*Y*) and the predicted variation of the model (*Q*^2^) approached 1.0. Thus, a highly reliable model was supported. To perform fuzzy k-means clustering, a principal component analysis (PCA) was first calculated across the 61 log transformed metabolomic measures of interest using the R “stat” package (R Development Core Team 2019). Three principal components were shown to explain ∼75% of the overall variability. The corresponding unstandardized scores were retained to calculate multivariate distances and perform the cluster analysis (Johnson 1998). Fuzzy k-means clustering was employed rather than other comparable techniques so that cluster membership could overlap, if necessary, using the “fclust” package in R. Silhouette index values and three-dimensional scatterplots were then utilized to help confirm the appropriate number of clusters.

Heatmaps were constructed using R routines for hierarchical clustering (R Development Core Team 2019). Heat map *Z*-scores were derived using the following steps. *1*) The average and standard deviation within a 25-min microdialysis sampling point were calculated across subjects from both the wakefulness and the isoflurane treatment groups. *2*) Raw metabolite intensities were then subtracted from the time point average and subsequently divided by that standard deviation. *3*) A time point *z*-score average was calculated separately for isoflurane and wakefulness subjects, which generated five data points per metabolite and treatment condition. *4*) Last, the *z*-score time point average from the isoflurane treatment condition was subtracted from the wakefulness treatment condition.

A volcano plot was generated using the Benjamini Hochberg adjusted *P* values (*q* values) and fold change. For each metabolite, the fold change was calculated using the median and dividing the median for isoflurane by the median for wakefulness. The median was used instead of the mean in order to provide a measurement of central tendency robust to the effects of outliers.

Using the Human Metabolome Database (HMDB), molecules were sorted into five chemical groups based on their closest direct/alternate parent compound: *1*) amino acids and analogs, *2*) nucleosides and analogs, *3*) organic acids and derivatives, *4*) monosaccharides and derivatives, and *5*) lipid and fatty acid derivatives. A final set of analyses quantified how the 21 metabolites with a fold change ≥ 4 and *q* value *P* < 0.0001 relate to the biochemical pathways in the Kyoto Encyclopedia of Genes and Genomes (KEGG) database for mice. Molecules commonly contribute to multiple biochemical pathways. Therefore, organizational criteria were developed to operationally define the relative percent contribution made by a measured molecule to major categories and subcategories of KEGG biofunction. First, the association between the measured molecule and the individual pathways was identified. Second, the frequency with which the molecule occurred in the pathways comprising the major and subcategories of biofunction was summed for each KEGG biofunction category. Third, these sums were expressed as the numerator relative to the total number of times the 21 molecules were identified in a KEGG pathway.

## RESULTS

### 

#### Microdialysis site localization.

All microdialysis samples were obtained from the PFC (Franklin and Paxinos 2008). Figure 1 summarizes the average location of the microdialysis sites for the 12 mice studied during wakefulness (Fig. 1*D*) and the 12 mice studied during anesthesia (Fig. 1*E*). Histological analyses of brains studied during wakefulness showed that mean (±SD) coordinates for dialysis probe placement in the PFC were 2.8 (±0.2) mm anterior, 1.3 (±0.2) mm lateral, and 1.1 (±0.1) mm ventral. Similar histological analyses of brains studied during anesthesia revealed mean (±SD) coordinates for dialysis probe placement in the PFC were 2.5 (±0.2) mm anterior, 1.3 (±0.2) mm lateral, and 1.1 (±0.1) mm ventral. The average probe location in Euclidian space for the waking group was within 0.3 mm of the probe location for the isoflurane group (Fig. 1, *D* and *E*).

#### Detection and identification of PFC metabolites.

Untargeted metabolomic analyses confirmed detection of 2,153 molecules, 91 of which were identified from a spectral list of more than 300 molecules with previously determined mass and retention time (Lu et al. 2010). Sixty-one of the 91 metabolites were identified during both wakefulness and isoflurane anesthesia (see Supplemental Table S1, at https://figshare.com/s/42669ca81d5079678004). Twenty-three molecules were measured only during wakefulness and seven molecules were identified only during anesthesia (see Supplemental Table S2, at https://figshare.com/s/104f004545d872edf924).

For the 61 molecules in Supplemental Table S1, the Fig. 2 heat maps use color to visualize differences in PFC metabolite levels during wakefulness and isoflurane anesthesia expressed as changes in *z*-score. The colors in each cell represent average ion intensity as a surrogate for relative molecule concentration. The numbers 1 through 5 on the abscissa of the heat maps correspond to the ordinal sequence of microdialysis samples, with 1 being the first and 5 being the final dialysis sample. The color distribution illustrates no systematic increase or decrease in analytes during the 125 min of microdialysis. Supplemental Table S1 provides the median, maximum, and minimum along with the *P* and *q* values for each molecule in Fig. 2.

#### Multivariate analyses.

**Fig. 3.**
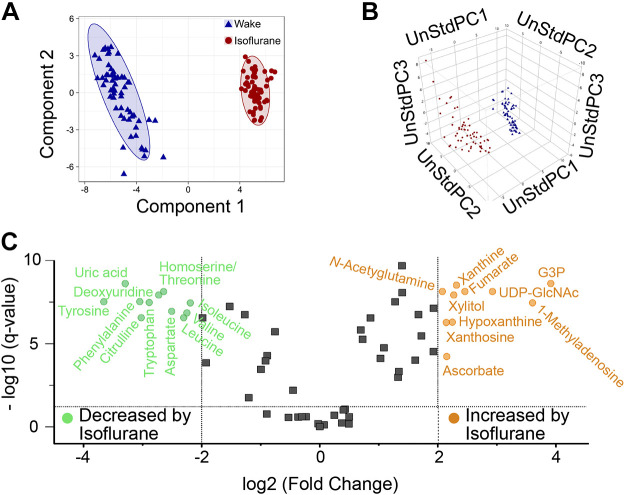
Summary of multivariate analyses for the 61 shared metabolites. *A*: each symbol in the partial least squares discriminate analysis (PLS-DA) score plot shows data from one dialysis sample collected during wakefulness (blue triangles, *n* = 60 samples) or isoflurane anesthesia (red circles, *n* = 60 samples). Ellipses define the 95% confidence interval for the state-space distribution of metabolites. *B*: fuzzy k-means cluster analysis on principal component analyses scores confirmed the PLS-DA distribution. The 120 points represent dialysis samples obtained during isoflurane anesthesia (red) or wakefulness (blue). Axes show unstandardized principle components (UnStdPC). *C*: volcano plot indicates fold change ≥ 4 and *q* < 0.0001 during anesthesia relative to wakefulness for molecules decreased by isoflurane (green, *n* = 11) or increased by isoflurane (orange, *n* = 10). Fold changes were calculated using median values in Supplemental Table S1 (https://figshare.com/s/42669ca81d5079678004). Points plotted in gray represent molecules (*n* = 40) that did not satisfy the *q* and fold change requirements.

The 61 shared metabolites were analyzed using three multivariate techniques: PLS-DA, PCA, and fuzzy k-means cluster analysis on log-transformed data. These analytic approaches all demonstrated a significant separation between the samples collected during wakefulness and during isoflurane anesthesia (Fig. 3). PLS-DA using two principle components accounted for ∼64% of the variance (Fig. 3*A*). Significant separation between states can be seen from the 95% confidence ellipses (*R*^2^ *X* = 0.466, *R*^2^ *Y* = 0.973, *Q*^2^ = 0.972). Fuzzy k-means clustering on three PCA scores validated the separation between wakefulness and isoflurane (Fig. 3*B*). Additionally, fuzzy k-mean values most strongly supported a two-cluster approach (Bezdek 1981; Johnson 1998). After identification and confirmation of two groupings of metabolites (wakefulness and isoflurane anesthesia), a combination approach was developed to investigate a subset of measures with the largest difference in magnitude between states. Both PLS-DA (Fig. 3*A*) and fuzzy k-means clustering technique (Fig. 3*B*) were separately used to evaluate the relationship between the 61 shared metabolites. Consequently, both analysis techniques resulted in similar determinations that the degree of separation between clusters was clear and corresponded to states of wakefulness and isoflurane anesthesia (Johnson 1998). A volcano plot (Fig. 3*C*) indicates the 21 shared metabolites having a fold change ≥ 4 and a significant Benjamini–Hochberg ANOVA *P* value, referred to as an adjusted *q* < 0.0001 (Supplemental Table S1). Of those 21 shared metabolites, 11 metabolites were decreased during isoflurane, and 10 metabolites were increased during isoflurane.

**Fig. 4.**
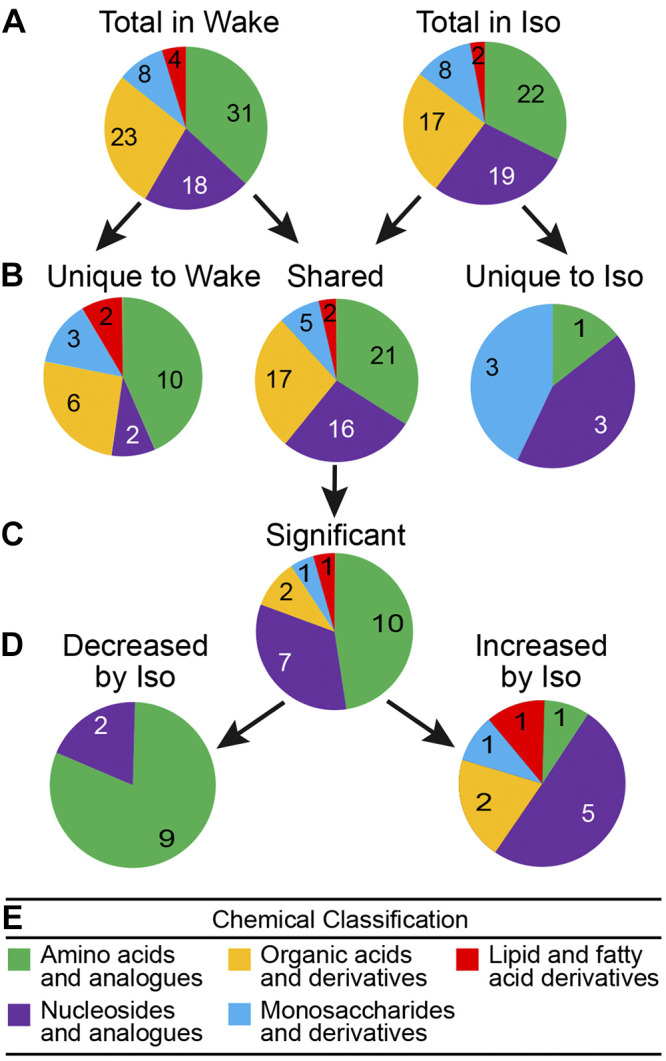
Pie charts schematize the general chemical taxonomy for all 91 molecules detected during wakefulness (Wake) and/or isoflurane anesthesia (Iso). Molecules were classified into 5 groups (see color key *E*) based on their closest direct/alternate parents in the Human Metabolome Database. Numbers of metabolites corresponding to the key categories are specified within each segment of the pie charts. *A* illustrates number and chemical classification of molecules measured during wakefulness (*left*, *n* = 84) and isoflurane anesthesia (*right*, *n* = 68). *B* divides the 91 metabolites into chemical classes for those detected only during wakefulness (*left*, *n* = 23), only during isoflurane anesthesia (*right*, *n* = 7), and during both wakefulness and anesthesia (*middle*, *n* = 61). For a list of all metabolites measured during both wakefulness and isoflurane anesthesia see Supplemental Table S1 (https://figshare.com/s/42669ca81d5079678004). For a list of metabolites unique to wakefulness and unique to anesthesia see Supplemental Table S2 (https://figshare.com/s/104f004545d872edf924). *C* indicates chemical taxonomy of the 21 shared metabolites that showed a fold change ≥ 4 and *q* < 0.0001 These molecules are identified in Fig. 5. *D* divides the 21 significant, shared metabolites into those that were decreased (*left*, *n* = 11) and increased (*right*, *n* = 10) during isoflurane.

Figure 4 categorizes the metabolites measured during wakefulness and/or isoflurane anesthesia into five chemical groups derived from the HMDB. The HMDB classification is based on the closest/alternate direct parent molecule. Figure 4*A* illustrates the number of metabolites measured in each chemical classification during wakefulness (Fig. 4*A* *left*) and during isoflurane anesthesia (Fig. 4*A* *right*). Figure 4*B* divides the 91 measured metabolites into three groups and indicates the number of metabolites in each chemical group for those metabolites measured only during wakefulness (Fig. 4*B* *left*), those measured only during anesthesia (Fig. 4*B* *right*), and those measured during both states (Fig. 4*B* *middle*). Figure 4*C* identifies the shared metabolites with levels that were significantly (*q* < 0.0001) decreased or increased by isoflurane. Figure 4*D* subdivides the 21 significantly changed molecules into the total number of molecules decreased or increased during isoflurane anesthesia.

#### Isoflurane anesthesia altered the PFC metabolome.

**Fig. 5.**
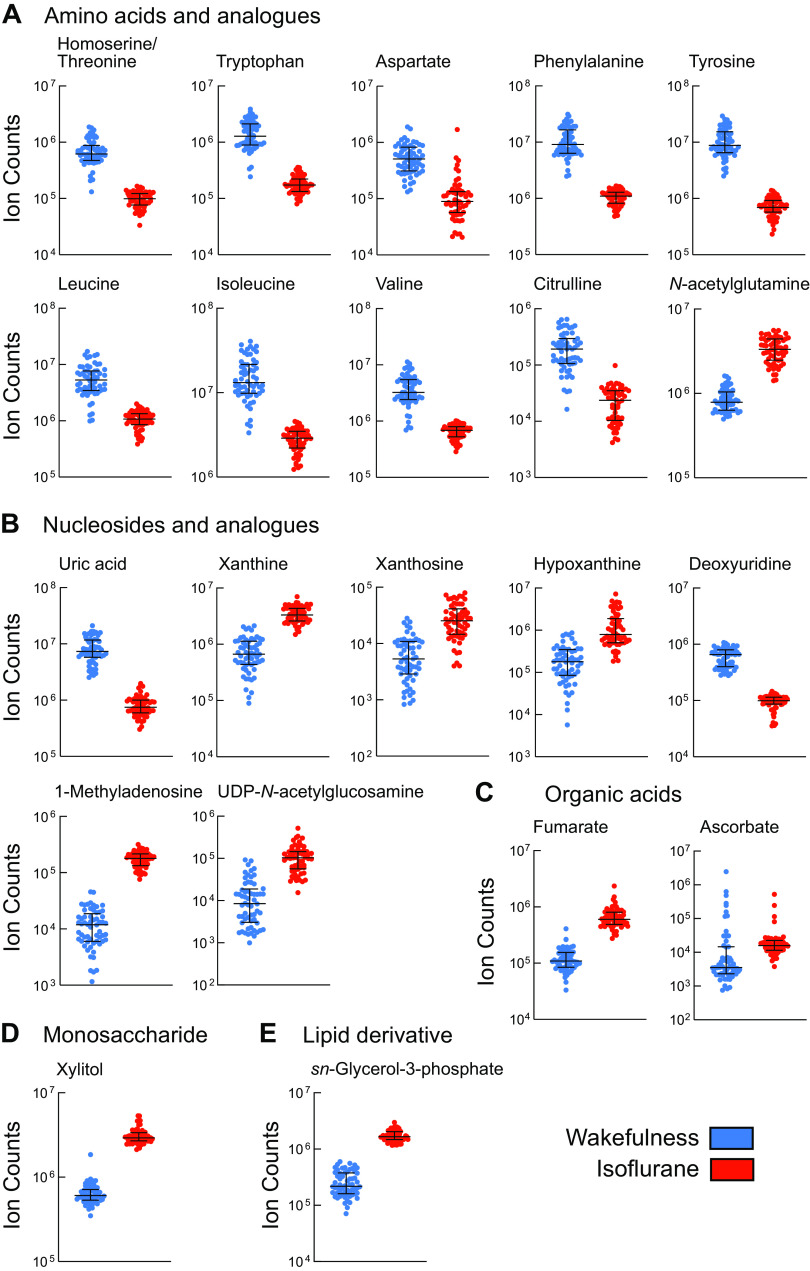
Median ± interquartile range showing relative concentration (raw intensity) of metabolites during wakefulness (blue) and isoflurane anesthesia (red). These metabolites (*n* = 21) all had a fold change ≥ 4 and *q* < 0.0001 (Supplemental Table S1, https://figshare.com/s/42669ca81d5079678004). *A*: amino acids and analogs. *B*: nucleosides and analogs. *C*: organic acids. *D*: monosaccharide. *E*: lipid derivative.

The Fig. 5 data are organized relative to the chemical taxonomy of the HMDB and pathways described by the KEGG database. Figure 5 shows individual plots of the 21 metabolites that had an isoflurane-induced fold change ≥ 4 and *q* < 0.0001. The most striking finding revealed by Fig. 5*A* is that, of the 10 amino acids satisfying these requirements, nine were decreased during isoflurane anesthesia. In contrast, the amino acid analog *N*-acetylglutamine was increased during administration of isoflurane. Figure 5*B* shows that five of the seven nucleosides and analogs were significantly increased during isoflurane anesthesia. The organic acids fumarate and ascorbate were both increased during isoflurane anesthesia (Fig. 5*C*). Xylitol was the only monosaccharide identified as significantly increased during anesthesia (Fig. 5*D*). The lipid derivative *sn*-glycerol 3-phosphate (G3P) also was significantly increased during isoflurane administration (Fig. 5*E*).

**Fig. 6.**
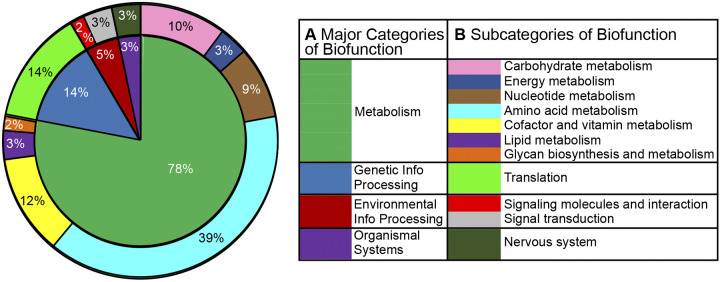
Major categories (*inner circle*) and subcategories (*outer circle*) of biofunction from Kyoto Encyclopedia of Genes and Genomes (KEGG) database. Circle plot represents the metabolites (*n* = 21) with levels that were significantly changed (fold change ≥ 4, *q* < 0.0001) during isoflurane anesthesia. Percentages within the plot indicate the total number of times the 21 molecules were identified in a KEGG pathway; e.g., molecules associated with amino acid metabolism comprised 23 of 59 pathway occurrences, or 39%. *A* shows the 4 major categories of biofunction illustrated by the *inner circle*; *B* lists 11 subcategories of biofunction identified by corresponding colors in the *outer circle*.

The 21 molecules with levels that were significantly altered during isoflurane anesthesia are grouped into their known biofunction pathways identified by the KEGG database (Fig. 6). Assigning the 21 molecules to known biofunction pathways identifies metabolic processes that were altered by isoflurane. The major biofunction pathway of metabolism (78%) contains three of the four pathways most affected during isoflurane anesthesia, including molecules contributing to amino acid metabolism (39%), metabolism of cofactors and vitamins (12%), and carbohydrate metabolism (10%). During anesthesia, all of the metabolites involved in genetic information processing (14%) were related to translation.

## DISCUSSION

The results revealed that molecules comprising the PFC metabolome were clustered into distinct groups that corresponded to states of wakefulness and isoflurane anesthesia. Cluster analysis of state-specific metabolite distributions (Fig. 3*A*) were confirmed by multivariate analyses (Fig. 3*B*). A strict criterion of a fourfold change and a *q* value <0.0001 was required for an analyte to be classified as either increased or decreased during anesthesia. Plotting the fold change produced a volcano plot (Fig. 3*C*) showing the metabolites that were significantly decreased or increased during isoflurane anesthesia. A taxonomic approach (Fig. 4) quantified the number of metabolites in chemical groups that were unique to wakefulness and unique to isoflurane anesthesia. The goals of comparing the PFC metabolome during states of wakefulness and anesthesia, and during sleep (Bourdon et al. 2018) and anesthesia, are discussed in the following subsections.

### 

#### Arousal state-specific changes in PFC metabolome.

Previous measures of the PFC metabolome during wakefulness and sleep identified 11 molecules that decreased significantly during sleep relative to wakefulness (Bourdon et al. 2018). Levels of those 11 molecules during isoflurane anesthesia relative to wakefulness revealed that only tryptophan significantly decreased during anesthesia (see Supplemental Table S3, at https://figshare.com/s/6ed8075f7ce12ea753eb). In contrast to sleep, during anesthesia there were significant increases in the levels of d-gluconate, glutamate, homovanillic acid, lactate, *N*-acetyl-β-alanine, *N*-acetylglutamine, succinate/methylmalonate, and uridine. Supplemental Table S3 reveals no change in levels of orotate and pyruvate. The lack of identity between the PFC metabolome during sleep and anesthesia is consistent with many studies indicating that sleep and anesthesia are distinctly different states (Akeju and Brown 2017; Baghdoyan and Lydic 2012; Garrity et al. 2015; Lydic and Baghdoyan 2005; Lydic et al. 2018; Vanini et al. 2020).

#### Isoflurane decreased amino acids and increased purines.

Tryptophan is an essential amino acid and a precursor for the biosynthesis of the sleep-modulating molecules serotonin and melatonin. Depending on brain region, loss of wakefulness is characterized by decreases in monoaminergic neurotransmission (Baghdoyan and Lydic 2012). Phenylalanine and tyrosine are precursors of dopamine, and both molecules decreased during isoflurane anesthesia (Fig. 5). Dopamine is a wakefulness-promoting neurotransmitter and a precursor of norepinephrine, which also promotes wakefulness (Baghdoyan and Lydic 2012). In rat PFC, dopamine increases during cortically activated episodes of wakefulness and REM sleep, in contrast to norepinephrine, which decreases during NREM and REM sleep compared with wakefulness (Léna et al. 2005). In rats, during continuous isoflurane anesthesia, administering inhibitors of dopamine and norepinephrine reuptake causes behavioral arousal and EEG activation (Solt et al. 2011). Optogenetic stimulation of dopamine neurons in mouse ventral tegmental area also causes arousal from isoflurane anesthesia (Taylor et al. 2016). Considered together, these findings are consistent with evidence that precursors of dopamine in the PFC promote wakefulness.

The branched-chain amino acids leucine, isoleucine, and valine (Fig. 5) were decreased during isoflurane anesthesia. Branched-chain amino acids are important for the synthesis of glutamate. Biosensor measurement showed that l-glutamate in PFC of B6 mice increased during extended episodes of wakefulness and decreased during NREM and REM sleep (Naylor et al. 2011). Amperometric detection of glutamate in rat PFC revealed decreases during NREM sleep and increases during cortically activated states of wakefulness and REM sleep (Dash et al. 2009). The foregoing results are consistent with metabolomic studies showing levels of PFC glutamate to be higher during wakefulness than during sleep (Bourdon et al. 2018). During isoflurane anesthesia there was also a significant decrease in citrulline and aspartate. The Fig. 5 results encourage future studies to determine whether isoflurane eliminates wakefulness, in part, by decreasing metabolic processes utilizing amino acids.

Figure 6*A* illustrates the percentage of measured molecules that subserve four major biofunctions defined by the KEGG database. Figure 6*B* shows that molecules involving amino acid metabolism comprised the largest biofunction subcategory (39%) that was altered during isoflurane anesthesia. The increase in *N*-acetylglutamine during isoflurane anesthesia (Fig. 5 and Supplemental Table S1) is of interest, considering evidence (Bourdon et al. 2018) that this molecule decreased during sleep relative to wakefulness (Supplemental Table S3). Protein synthesis can be increased or decreased by volatile anesthetics (Fütterer et al. 2004), and the present results show that levels of many molecules involved in translation changed during isoflurane anesthesia relative to wakefulness (Fig. 6).

The purine derivative 1-methyladenosine was significantly increased during isoflurane anesthesia (Fig. 5 and Supplemental Table S1). This finding is consistent with recent evidence that one of the major predictors of isoflurane anesthesia was adenosine concentration in mouse PFC (Zhang et al. 2020). Meta-analyses indicate that all types of anesthesia increase adenosine (van der Mierden et al. 2018). The mechanisms by which 1-methyladenosine increased during isoflurane anesthesia are not known. The KEGG database indicates that xanthosine and hypoxanthine, through separate pathways, are direct precursors of xanthine. All three molecules were increased during isoflurane anesthesia. Interestingly, the first product of xanthine metabolism, uric acid, was significantly decreased during isoflurane anesthesia.

#### PFC lipid and carbohydrate metabolites.

Figure 5 shows that the lipid precursor *sn*-glycerol 3-phosphate significantly increased during isoflurane anesthesia. G3P is an essential synthetic component of energy metabolism/storage (glycerolipids), and G3P is a constituent of lipid rafts within the cell membrane. Anesthetics including isoflurane disrupt lipid rafts and, via a phospholipase D_2_-dependent mechanism, activate two-pore potassium channels (TWIK-related K^+^ channel 1, TREK-1), which underlie a membrane-mediated mechanism for volatile anesthetics (Pavel et al. 2020). Autoradiographic data confirm densely distributed TREK-1 mRNA in mouse PFC (Maingret et al. 2000).

Carbohydrate metabolism was inhibited by isoflurane, which may explain the significantly increased levels of uridine diphosphate-*N*-acetylglucosamine (UDP-GlcNAc), fumarate, ascorbate, and xylitol during isoflurane anesthesia (Fig. 5 and Supplemental Table S1). The hexosamine biosynthetic pathway converts small amounts of glucose into UDP-GlcNAc. Additionally, UDP-glucose (Supplemental Table S2) was uniquely detected during isoflurane anesthesia. UDP-GlcNAc was significantly increased during isoflurane anesthesia (Fig. 5). The mechanisms causing UDP-GlcNAc to be significantly increased during isoflurane anesthesia are not known. UDP-GlcNAc serves as a donor molecule to transfer GlcNAc to proteins (O-GlcNAcylation). Within mouse PFC, levels of O-GlcNAcylation promote inhibitory synaptic transmission (Cho et al. 2020). This raises the question of whether O-GlcNAcylation contributes to the inhibitory action of volatile anesthesia.

#### Limitations and interpretations.

Metabolomic studies confront a number of analytic limitations (Alonso et al. 2015), acknowledged previously (Bourdon et al. 2018). The present study using anesthesia as a tool for eliminating wakefulness was limited to one concentration of isoflurane. Using the same isoflurane concentration as was used in our targeted study (Zhang et al. 2020) enabled comparisons between effects of isoflurane on PFC metabolome and PFC neurotransmitters (Zhang et al. 2020). The spatial and temporal resolving power of microdialysis (Watson et al. 2006) limits the ability to address the anatomic (Carlén 2017; van Heukelum et al. 2020) and functional (Xing et al. 2020) subregions that exist within the PFC.

In conclusion, the present results reveal significant differences during wakefulness and isoflurane anesthesia in levels of molecules within the PFC. The results support three additional interpretations. First, a large number of precursor molecules (Supplemental Table S1) and neurotransmitters (Zhang et al. 2020) are associated with the loss of wakefulness during isoflurane anesthesia. Second, the present finding that states of consciousness are modulated by many molecules in the PFC is consistent with anatomic evidence (Furster 2015) that the PFC influences chemical transmission in multiple brain regions. For example, delivery of adenosine antagonists to mouse PFC promotes arousal by increasing ACh release in the PFC and in the pontine reticular formation (Van Dort et al. 2009). Third, systems biology offers specific tools for elucidating the complexity of interacting neurochemical networks. Large metabolomic data sets (Supplemental Table S1) can unmask network relationships (Fig. 5) that are not apparent in reductionistic measures of any single molecule. Furthermore, large data sets inform targeted approaches designed to test novel hypotheses. The present results are a product of such a discovery cycle. Untargeted approaches previously revealed changes in the PFC metabolome during the loss of wakefulness caused by sleep (Bourdon et al. 2018). Those findings prompted a hypothesis-directed study revealing the reorganization among eight PFC neurotransmitters during the isoflurane-induced loss of wakefulness (Zhang et al. 2020). The neurotransmitter data encouraged the present use of anesthesia as a tool for eliminating wakefulness, making it possible to compare the PFC metabolome during anesthesia and sleep (Bourdon et al. 2018). The present and previous results (Bourdon et al. 2018; Zhang et al. 2020) indicate that the loss of wakefulness initiated by sleep or anesthesia is not a function of any single, endogenous molecule. The present omics data support the systems biology perspective (Karakitsou et al. 2019) that higher-level phenotypes, such as states of consciousness, are emergent processes reflecting dynamic interactions between anatomically distributed neuronal networks and a humbling number of molecules.

## GRANTS

This work was supported by Departments of Anesthesiology and Psychology, University of Tennessee, Knoxville, TN, and National Heart, Lung, and Blood Institute Grant HL-65272 (R.L.).

## DISCLAIMERS

This paper has been coauthored by UT-Battelle, LLC, under Contract No. DE-AC05-00OR22725 with the U.S. Department of Energy. The United States Government retains and the publisher, by accepting the article for publication, acknowledges that the United States Government retains a nonexclusive, paid-up, irrevocable, worldwide license to publish or reproduce the published form of this paper, or allow others to do so, for United States Government purposes. The Department of Energy will provide public access to these results of federally sponsored research in accordance with the DOE Public Access Plan (https://energy.gov/downloads/doe-public-access-plan).

## DISCLOSURES

No conflicts of interest, financial or otherwise, are declared by the authors.

## AUTHOR CONTRIBUTIONS

H.A.B. and R.L. conceived and designed research; A.G.B. and A.K.B. performed experiments; A.G.B., A.K.B, J.M.P., S.R.C., H.A.B., and R.L. analyzed data; A.G.B., A.K.B, J.M.P. S.R.C., D.A.J, H.A.B., and R.L. interpreted results of experiments; R.L., H.A.B., A.G.B., A.K.B., J.M.P., D.A.J., and S.R.C. edited and revised manuscript; A.G.B., A.K.B, J.M.P., S.R.C., D.A.J., H.A.B., and R.L. approved final version of manuscript.
